# Risk Assessment for Corneal Ectasia following Photorefractive Keratectomy

**DOI:** 10.1155/2017/2434830

**Published:** 2017-07-26

**Authors:** Nir Sorkin, Igor Kaiserman, Yuval Domniz, Tzahi Sela, Gur Munzer, David Varssano

**Affiliations:** ^1^Department of Ophthalmology, Tel Aviv Sourasky Medical Center, Affiliated to the Sackler School of Medicine, Tel Aviv University, Tel Aviv, Israel; ^2^Care Laser Inc., Tel Aviv, Israel; ^3^Department of Ophthalmology, Barzilai Medical Center, Ashkelon, Israel; ^4^Faculty of Health Sciences, Ben-Gurion University of the Negev, Beersheba, Israel

## Abstract

**Purpose:**

To analyze the risk factors associated with a series of ectasia cases following photorefractive keratectomy (PRK) and all published cases.

**Methods:**

In a retrospective study on post-PRK ectasia patients, 9 eyes of 7 patients were included, in addition to 20 eyes of 13 patients from the literature. Risk of post-PRK ectasia was calculated using the ectasia risk score system (ERSS) for laser in situ keratomileusis (LASIK) patients. The percent tissue altered (PTA) was also evaluated.

**Results:**

ERSS scoring of zero for age, RSB, and spherical equivalent was found in 66%, 86%, and 86% of the eyes, respectively. Pachymetry risk score was 2 in 60% of the eyes and 3 or 4 in 16% of the eyes. Topography risk score was 3 in 41% of the eyes and 4 in 21% of the eyes. Cumulative ectasia risk score was ≥4 (high risk) in 77% of the eyes and ≥3 (medium and high risk) in 86% of the eyes. Average PTA was 23.2 ± 7.0%. All eyes but one had a PTA < 40%.

**Conclusions:**

Preoperative corneal topographic abnormalities and thin corneas may be significant risk factors for developing ectasia following PRK. Post-LASIK ectasia risk scoring also has relevance in the risk for developing post-PRK ectasia.

## 1. Introduction

Corneal ectasia is a well-recognized, serious complication of laser in situ keratomileusis (LASIK) [1]. It is characterized by progressive stromal thinning and steepening of the cornea, resulting in refractive aberrations and vision loss. The incidence in LASIK patients is estimated to be between 0.04% and 0.6% [1]. Ectasia following PRK is considered extremely rare and has been described previously in only a few case reports [2–8] and small case series [9–12].

The risk for developing post-LASIK ectasia is routinely assessed prior to surgery and includes evaluation of patient age, degree of refractive correction, topographic findings, corneal pachymetry, and residual stromal bed (RSB). In 2008, Randleman et al. [13] proposed an ectasia risk score system (ERSS) for evaluating post-LASIK ectasia risk [13] and later validated it in a second study [14]. More recently, the value of percent tissue altered (PTA) was found to be a robust indicator of the risk for post-LASIK ectasia [15, 16]. Due to the paucity of post-PRK ectasia cases, there is no corresponding system for evaluating its risk of occurrence.

In this study, risk factors for development of post-PRK ectasia were analyzed in a series of post-PRK ectasia cases, and in all post-PRK ectasia cases found in the literature.

## 2. Materials and Methods

This retrospective cohort study of post-PRK corneal ectasia patients was approved by the Institutional Review Board of the Tel Aviv Sourasky Medical Center, Tel Aviv, Israel, and followed the tenets of the Declaration of Helsinki.

### 2.1. Patients

Cases that were 18 years and older with a history of PRK and a diagnosis of corneal ectasia were included in the study. None had any previous ocular pathology or relevant ocular or familial history. Our series consisted of 9 eyes of 7 patients (study cohort) who developed post-PRK ectasia, out of 31,045 eyes (0.029%) who underwent PRK during the same time period (2004–2015). There were 4 males and 3 females, mean age 26 ± 8.2 years (range 18–39 years), 5 right eyes and 4 left eyes. The 8 papers [5–12], published between 2000 and 2008, which were included in the literature analysis, reported a total of 20 eyes of 13 patients with post-PRK ectasia (literature search eyes).

### 2.2. Methods

Data were collected from the electronic medical database of Care Vision, a refractive surgery facility in Tel Aviv, Israel. The diagnosis of post-PRK ectasia was verified by a cornea surgeon (DV). Ectasia was defined as progressive steepening of the central or mid-lower cornea, and/or progressive thinning of the central or mid-lower cornea. When evaluating for ectasia, the examiner (DV) took into account changes in manifest refraction, keratometry, pachymetry, inferior-superior differences, location of the thinnest point and steepest point, and posterior elevation (thinnest point and posterior elevation were available in cases where Scheimpflug or slit-scanning tomography had been performed). Topography was obtained from the TMS (Tomey, Tennenlohe, Germany), Orbscan (Bausch & Lomb, Rochester, NY, USA), Topolyzer (Oculus, Wetzlar, Germany), and Sirius (C.S.O., Florence, Italy). Preoperative pachymetry was obtained using ultrasound, Orbscan (Bausch & Lomb, Rochester, NY, USA), or Sirius (C.S.O., Florence, Italy). RSB was calculated based on the formula

(1) $$\begin{array}{c} RSB=preoperative\;pachymetry-ablation\;depth-50\;\mu m\;\left(estimated\;epithelial\;thickness\right). \end{array}$$

### 2.3. Main Outcome Measures

The following data were recorded and assessed: patient demographics; ocular and familial history; preoperative data including best spectacle-corrected visual acuity (BSCVA), manifest refraction, pachymetry and topography; and perioperative and postoperative data including ablation depth, RSB (calculated), number of enhancement procedures, and the time from PRK until the appearance of ectasia.

A systematic search of the literature was carried out on August 25, 2015, using the PubMed search engine and the search terms *(Photorefractive Keratectomy* OR *PRK)* AND *ectasia*. All case reports and case series that had data on topography, and either RSB, pachymetry, or refractive error, were included. Cases without topography data were excluded. In cases where topography images were available, the diagnosis of post-PRK ectasia was verified by a cornea surgeon (DV). In cases where topography data was available without topography images, the data was recorded as detailed by that paper's author. The same patient data, as detailed above, were collected from all published cases, where present (in cases where partial data was available, only the available data was recorded).

There were 15 papers in the literature describing post-PRK ectasia: 7 case reports [2–8], 4 case series [9–12], and 4 large series composed mainly of post-LASIK ectasia patients and 1 to 3 post-PRK ectasia patients in each [17–20]. The 4 large post-LASIK series that included a few post-PRK patients did not present separate data on the post-PRK eyes and therefore were not included in the analysis. Three of the case reports [2–4] did not include preoperative topography data and were therefore excluded. One case series [11] only had topography data for 2 of 8 eyes, and therefore, only those 2 eyes were included. The 8 papers that were included reported a total of 20 eyes of 13 patients: 7 eyes of 4 patients in 7 case reports and 13 eyes of 9 patients in 4 case series.

Risk factors for ectasia following PRK were those of the ERSS (Table 1) [13] and the calculated PTA [15, 16].

PTA for PRK was calculated based on the formula

(2) $$\begin{array}{c} PTA=\frac{\left(ablation\;depth+50\;\mu m\;for\;epithelial\;thickness\right)}{preoperative\;pachymetry}. \end{array}$$

## 3. Results

**Figure 1:**
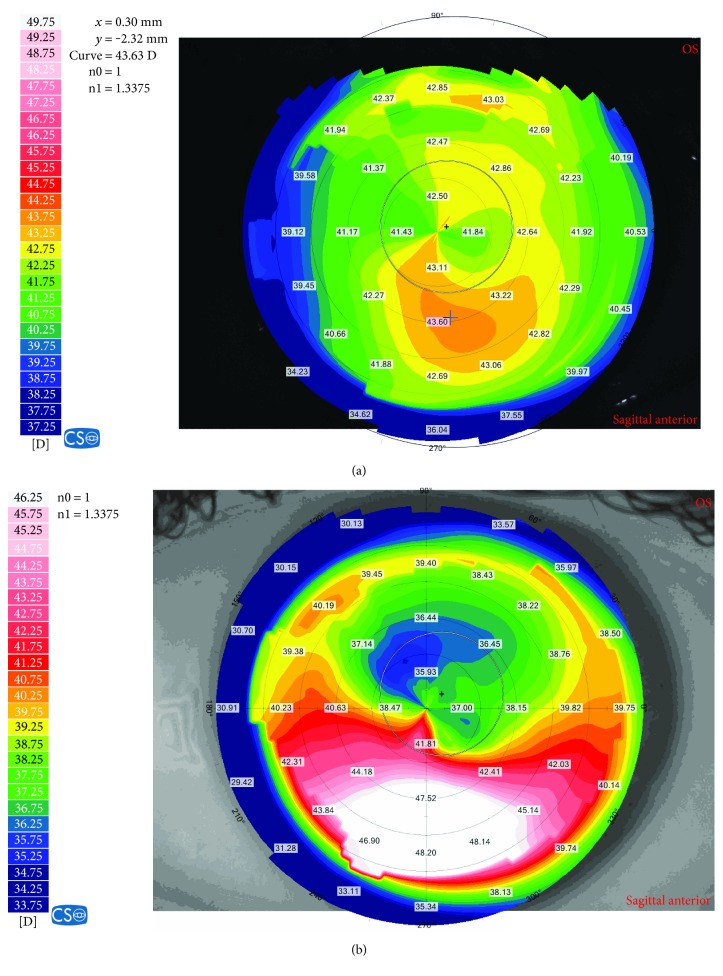
The preoperative (a) and post-ectasia (b) topographies of case number 7 in Table 2.

In our series, there were 9 eyes of 7 patients. Refractive error was low to moderate (SEQ range −0.75 to −3.88 D). None of the patients had any refractive enhancements following the PRK procedure. All 9 eyes had abnormal preoperative topographic patterns, according to the ERSS system [13], including asymmetric bowtie (ABT), skewed radial axis (SRAX), or abnormal inferior-superior value (I-S). Two of 7 patients developed bilateral ectasia and 5 of 7 patients developed unilateral ectasia. In the patients who developed unilateral ectasia, preoperative topography of the fellow eyes also showed topographic abnormalities, including two eyes with ABT (case numbers 2 and 7), two eyes with SRAX (case numbers 1 and 6), and one eye with I-S value of 1.5 (case number 3). Pachymetry prior to surgery was below 500 *μ*m in 3 of the eyes. Maximal ablation depth was 52 ± 18 *μ*m (range 23–74). Calculated RSB was below 400 *μ*m in 3 of the eyes. Demographic, preoperative, and postoperative data of our series are shown in Table 2.  Figure 1 shows the preoperative (Figure 1(a)) and post-ectasia (Figure 1(b)) topography of one of the patients (case number 7 in Table 2).

Ectasia was diagnosed 27.2 ± 12.4 months (range 3.3–45) following surgery. Ectasia was diagnosed earlier than 12 months following PRK in one eye. At the time of ectasia diagnosis, all eyes that had preoperative posterior corneal elevation values showed substantial increase of those values from 32 ± 7 *μ*m (range 22–40) to 51 ± 15 *μ*m (range 31–79) (Table 2). In addition, 8 of 9 eyes had substantial progression of inferior steepening, as demonstrated by the increase in I-S values (Table 2). One eye had no change in I-S value, but had progression of posterior corneal elevation with an inferior displacement of the posterior elevation apex (case number 3, Table 2). Only 1 of 9 eyes showed substantial alterations in manifest refraction and BSCVA at the time of ectasia diagnosis (Case no. 7, Table 2). Central keratometric values did not show a substantial change at the time of ectasia diagnosis. Following the diagnosis of ectasia, 5 of 9 eyes underwent corneal crosslinking and had no progression during the remaining follow-up. Four of 9 eyes were monitored and showed no evidence of progression. None of the eyes required keratoplasty.

Table 3 summarizes the demographic, preoperative, and postoperative data of the 20 literature search eyes that were included in the analysis.

The risk factors for the development of ectasia in all eyes, including the study eyes and the published cases found in the literature search, were analyzed using the ERSS criteria [13] and are summarized in Table 4.

**Figure 2:**
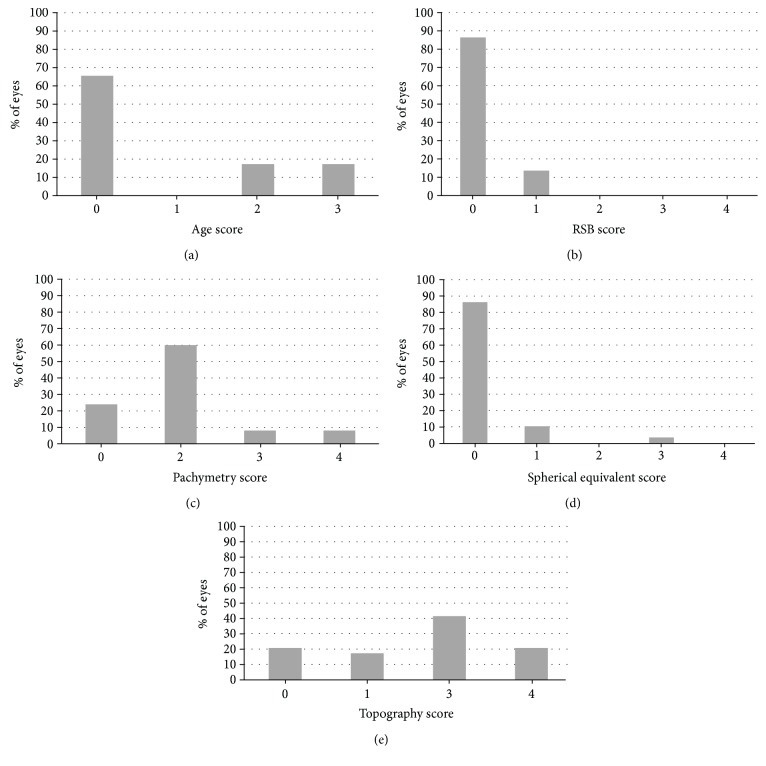
Distribution of the eyes by risk groups (included were eyes from the study cohort and eyes from the literature search): (a) age, (b) residual stromal bed (RSB), (c) pachymetry, (d) spherical equivalent, (e) topography.

The distribution of all eyes (including eyes from the study cohort and eyes from the literature search) by risk groups is presented in Figure 2 and Table 5.

**Figure 3:**
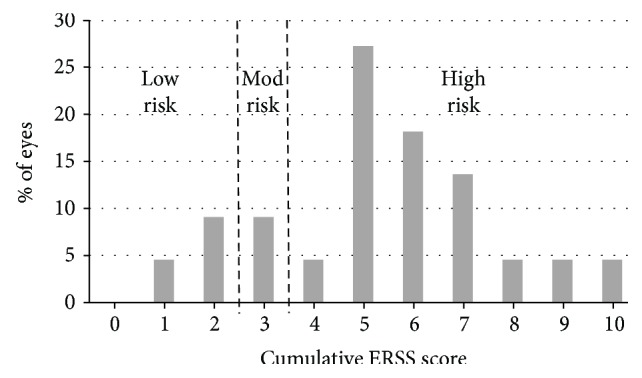
Distribution of the cumulative ectasia risk scores. Included in the analysis were 22 eyes (from the study cohort and from the literature search) that had available data for all ERSS (ectasia risk score system) parameters.

The mean cumulative ectasia risk score was 5.5 ± 2.3, with a range of 1 to 10 of 19 possible points. Cumulative ectasia risk score of 4 or greater, defined as high risk [13], was present in 77% of the eyes; 14% of the eyes were classified as low risk (2 points or less), and 9% as moderate risk (3 points). The distribution of the cumulative risk scores (for all eyes, including the study cohort and eyes from the literature search) is presented in Figure 3.

PTA was 20.1 ± 3.4% (range 14.6–25.2) in the study cohort, and 26.6 ± 7.7% (range 16.2–40.3) in the literature search eyes. PTA of all eyes was 23.2 ± 7.0% (range 14.6–40.3). All eyes had a PTA < 40%, except for one literature-search eye with a PTA of 40.3%.

## 4. Discussion

Post-PRK ectasia is much less prevalent than post-LASIK ectasia. In the study cohort, the rate of post-PRK ectasia was 9 out of 31,045 eyes (0.029%) who underwent PRK during the same period. This is compared with 0.1% to 0.66% reported following LASIK [1].

Ectasia was diagnosed postoperatively at 27.2 ± 12.4 months (range 3.3–44.8) in our cohort. Data in the literature on post-LASIK ectasia show ectasia diagnosis as early as 1 week and as late as 48 months [13]. However, most cases of post-LASIK ectasia are diagnosed during the first year [13]. In our series, only one eye (11%) had been diagnosed with post-PRK ectasia during the first postoperative year. Among the literature search eyes, 8 of 20 (40%) have been diagnosed during the first year. Altogether, the rate of first-year ectasia diagnosis in PRK patients was 31%. This may indicate that post-PRK ectasia is diagnosed later than post-LASIK ectasia, although this cannot be determined conclusively, due to the small sample size of post-PRK ectasia cases and the large variability of diagnosis time.

At the time of ectasia diagnosis, most of the patients (8 of 9 eyes) had no substantial change in central keratometry, manifest refraction, or BSCVA, despite having significant topographic changes, as evident in the increase in I-S values. This indicates early-stage diagnosis that was made during routine follow-up, while the patients were still asymptomatic. Therefore, it may be important to maintain long-term annual follow-up of PRK patients. None of the eyes in our cohort required keratoplasty. All cases were managed by either crosslinking or monitoring for progression. Both options should be considered in such cases. The initial choice of management should be based on the degree of refractive and topographic progression, and ongoing monitoring may be crucial.

In their validation of the ERSS criteria, Randleman et al. [14] reevaluated the 5 risk factors for ectasia following LASIK: abnormal preoperative topographic patterns, low RSB thickness, young age, low preoperative corneal pachymetry, and high myopia. In their study, abnormal preoperative topography was the most significant risk factor: 59.6% of the eyes had a score of 3 (14.7%) or 4 (44.9%). In our study, 62.1% of the eyes had a topography score of 3 (41.4%) or 4 (20.7%), documenting a dominance of suspicious abnormal preoperative topographic patterns in post-PRK ectasia patients.

In Randleman's analysis, RSB was the next risk factor in order of significance. Eighty-six percent of our post-PRK patients had RSB scores of zero in contrast to the 31.6% in Randleman's analysis. This difference may be a direct result of the lack of a stromal flap in PRK, which results in thicker RSB. Thus, the RSB score of the ERSS may not be relevant in PRK patients.

The age and SEQ scores of zero (65.6% and 86.2%, resp.) in the vast majority of the study cohort may indicate less influence of those factors on the risk of ectasia. These values are very similar to those reported in the validation study of the ERSS criteria [14], in which age and SEQ score of zero were found in 68.4% and 72.8% of the patients, respectively.

Preoperative corneal thickness was the least significant risk factor in the ERSS [13], but significant in our study: 60.0% had a score of 2 (low risk) and 16.0% had a score of either 3 (medium risk) or 4 (high risk). These scores may signify the importance of preoperative corneal thickness in the evaluation of the risk for post-PRK ectasia.

The majority (77%) of eyes included in the analysis were at high risk for ectasia according to the ERSS. In the initial ERSS publication containing the original scoring system, 92.6% of post-LASIK ectasia eyes were at high risk. Thus, using ERSS to evaluate risk for posttreatment ectasia in PRK may have value.

Average PTA was low at 23.2 ± 7.0%. Lower PTA values are expected following PRK, since there is no creation of a LASIK flap and therefore less manipulation of the anterior stroma. Regarding PRK, there is no data as of yet regarding the PTA threshold that may indicate higher risk for post-PRK ectasia. In eyes with normal preoperative topographies, PTA ≥ 40% was previously found to be a substantial risk factor for post-LASIK ectasia [15]. A study on post-LASIK ectasia eyes compared average PTA values between eyes with normal, low-suspect, and high-suspect preoperative topographies. The results showed average PTA of 45, 39, and 36% in each of the groups, respectively [16]. This leads to the conclusion that in eyes with preoperative topographic abnormalities, less manipulation of stroma is needed to induce ectasia. Only two eyes (both in the literature search group) had a PTA higher than 36% (values of 38.0% and 40.3%). This may indicate that high-risk topographic abnormalities are more indicative of post-PRK ectasia risk than PTA.

There are certain limitations to this study. Because this was a retrospective study and because post-PRK ectasia is rare, the data available were limited, which limited the number of cases that could be analyzed. Not all required data was available for the literature eyes. Also, since the period that was evaluated spanned 15 years, measurements (such as pachymetry, keratometry, and topography) were obtained by different devices. This affects the accuracy of comparison. The geographic distribution of included post-PRK ectasia cases probably does not properly represent the true global distribution of post-PRK ectasia due to a probable under-reporting of post-PRK ectasia worldwide. The presence of a large group originating from a single center may reflect local factors that cannot be well extrapolated to PRK candidates elsewhere.

In conclusion, while there is similarity between the risk factors for ectasia following LASIK and PRK, the factors differ in importance for each of the procedures. The findings demonstrate that ERSS, the risk score system developed and validated for LASIK, may have value in PRK ectasia risk evaluation. This emphasizes the need for a dedicated scoring system in PRK, despite the rarity of ectasia following PRK. It goes without saying that until such a scoring system is developed, increased caution must be exercised when abnormal values exist.

## Figures and Tables

**Table 1:** The ectasia risk score system (ERSS) for ectasia following LASIK^a^ [13].

Parameter	Points				
	4	3	2	1	0
Topography	FFKC^b^	Inf. steep.^c^	—	ABT^d^	Normal/SBT^e^
RSB^f^ (*μ*m)	<240	240 to 259	260 to 279	280 to 299	>300
Age (years)	—	18 to 21	22 to 25	26 to 29	>30
Pachymetry (*μ*m)	<450	451 to 480	481 to 510	—	>510
SEQ^g^ (D)	>−14	>−12 to −14	>−10 to −12	>−8 to −10	≤−8
Low risk: 0–2 points Moderate risk: 3 points High risk: ≥4 points					

^a^Laser-assisted in situ keratomileusis; ^b^forme fruste keratoconus; ^c^inferior steepening; ^d^asymmetric bowtie; ^e^symmetric bowtie; ^f^residual stromal bed; ^g^manifest refraction spherical equivalent.

**Table 2:** Study cohort—demographic and pre- and postoperative data.

Case number	Age	Sex	Eye number	Eye	Preoperative												Postoperative—time of ectasia diagnosis										
					Pachy^a^ (*μ*m)	Ablation depth (*μ*m)	Calc. RSB^b^ (*μ*m)	Sph^c^ (D)	Cyl^d^(D)	SEQ^e^ (D)	BSCVA^f^ (Snellen)	Anterior corneal topo^g^	Ks^h^	Kf^i^	Ave K^j^	Posterior elevation (*μ*m)	Time to onset of ectasia (months)	Pachy (*μ*m)	Sph (D)	Cyl (D)	SEQ (D)	BSCVA (Snellen)	Anterior corneal topo	Ks	Kf	Ave K	Posterior elevation (*μ*m)
1	21	M	1	R	441	44	347	−2.00	0.00	−2.00	1.2	ABT^k^ SRAX^l^	45.0	44.5	44.7	—	44.8	398	+2.00	−1.75	+1.13	1.0	I-S ~7.5	42.1	40.4	41.3	31
2	25	M	2	R	481	71	360	−3.50	−0.75	−3.88	1.0	ABT	47.0	46.6	46.8	—	3.3	390	−1.00	0.00	−1.00	1.2	I-S ~3.0	44.0	43.5	43.8	—
3	33	F	3	R	496	45	401	−2.50	−0.50	−2.75	1.0	I-S^m^ ~1.5	46.8	45.4	46.1	39	22.5	431	−0.75	0.00	−0.75	1.0	I-S ~1.5	45.2	44.4	44.8	45
4	18	M	4	R	535	52	433	−2.50	−1.00	−3.00	1.3	I-S ~2	45.8	44.8	45.3	26	33.3	452	0.25	0.00	0.25	1.0	I-S ~3.0	43.3	42.3	42.8	44
			5	L	538	63	425	−3.00	−1.25	−3.63	1.3	I-S ~2	45.9	44.7	45.3	29	33.3	444	0.00	0.00	0.00	1.0	I-S ~3.5	42.5	42.1	42.3	47
5	21	M	6	R	500	23	427	−0.75	0.00	−0.75	1.0	ABT	43.5	42.8	43.2	39	28.4	460	0.00	0.00	0.00	0.9	I-S ~2.1	42.8	41.9	42.4	45
			7	L	504	29	425	−1.00	−0.50	−1.25	1.0	ABT	43.7	42.5	43.1	40	28.4	435	0.00	−1.50	−0.75	0.9	I-S ~5.3	43.3	40.9	42.1	65
6	39	F	8	L	552	74	428	−2.50	−1.75	−3.38	1.0	SRAX	44.0	42.5	43.3	28	36.5	411	0.25	−1.00	−0.25	1.0	I-S ~2.7	42.5	40.7	41.6	55
7	24	F	9	L	514	67	397	−3.00	−1.00	−3.50	1.0	I-S ~1.3 SRAX	43.4	42.2	42.8	22	14.5	450	1.00	−2.50	−0.25	0.5	I-S ~10.5	40.3	37.6	38.9	79

Mean	26.2	—	—	—	507	52	405	−2.31	−0.75	−2.68	—	—	45.0	44.0	44.5	32	27.2	430	+0.19	−0.75	−0.18	—	—	42.9	41.5	42.2	51
SD	8.2	—	—	—	34	18	32	0.92	0.57	1.11	—	—	1.4	1.5	1.5	7	12.4	25	0.89	0.97	0.64	—	—	1.4	2.0	1.6	15
Min	18	—	—	—	441	23	347	−3.50	−1.75	−3.88	1.0	—	43.4	42.2	42.8	22	3.3	390	−1.00	−2.50	−1.00	0.5	—	40.3	37.6	38.9	31
Max	39	—	—	—	552	74	433	−0.75	0.00	−0.75	1.3	—	47.0	46.6	46.8	40	44.8	460	+2.00	0.00	+1.13	1.2	—	45.2	44.4	44.8	79

^a^Pachymetry; ^b^calculated residual stromal bed; ^c^sphere; ^d^cylinder; ^e^spherical equivalent; ^f^best spectacle-corrected visual acuity; ^g^topography; ^h^steep keratometry; ^i^flat keratometry; ^j^average keratometry; ^k^asymmetric bowtie; ^l^skewed radial axis; ^m^inferior-superior value.

**Table 3:** Literature review—demographic and pre- and postoperative data.

Eye number	Reference year	Number of eyes	Number of pts.	Age (years)	Sex	Eye	Preoperative refraction				Topographic findings	Measured pachy^c^ (*μ*m)	Ablation depth (*μ*m)	Calculated RSB^d^ (*μ*m)	Retreat^e^	Time to onset of ectasia (months)	Family history	Ocular history
							Sphere (D)	Cylinder (D)	BSCVA^a^ (Snellen)	SEQ^b^ (D)								
1	2000 [12]	2	1	41	F	R	−4.00	−3.50	0.7	−5.75	KCN^f^ or FFKCN^g^	—	—	—	1	—		
2						L	−5.50	−2.50	0.7	−6.75	KCN or FFKCN	—	—	—	1	—		

3	2002 [11]	2	2	34	M	L	−12.00	−1.50	0.5	−12.75	Normal	—	144	—	1	46		Amblyopia
4				39	M	R	−3.00	−4.50	0.3	−5.25	KCN	—	—	—	0	10		KCN

5	2006 [6]	2	1	22	M	R	−1.50	−1.00	1.0	−2.00	D sign	495	32	413	0	52		
6						L	−1.50	−1.00	1.0	−2.00	SRAX^h^ −60° I-S^i^ −0.9 (susp. FFKCN)	495	30	415	0	52		

7	2006 [13]	4	2	37	M	R	−1.50	−2.50	1.0	−2.75	I-S ~3	472	53	369	0	0.25		
8						L	−4.00	−3.00	0.7	−5.50	Central steep.^j^	441	100	291	0	0.25		
9				40	M	R	−4.75	−3.75	1.0	−6.63	SBT^k^	509	98	365^∗^	0	10	Sister had bil. PK^l^	Eye rubber
10						L	−5.25	−4.00	1.0	−7.25	SBT	508	106	356^∗^	0	1.5		

11	2006 [9]	2	1	42	M	R	−9.25	−1.25	0.8	−9.88	SBT	477	142	285	—	18		
12						L	−8.25	−2.00	0.8	−9.25	SBT	481	133	298	—	18		

13	2007 [10]	5	4	38	F	R	−7.00	−3.00	—	−8.50	Inf. steep.^m^	520	—	—	0	36		
14						L	−6.00	−4.00	—	−8.00	Inf. steep.	510	—	—	0	36		
15				31	M	R	−4.50	−1.75	1.0	−5.38	SBT	487	—	—	0	13		LE KCN s/p PK^n^
16				31	M	L	−3.75	−0.50	1.0	−4.00	Inf. steep., SRAX	492	91	351	0	30		
17				38	F	L	−1.50	−1.25	1.0	−2.13	Inf. Steep.	509	50	409	0	4		

18	2007 [8]	2	1	35	M	R	−3.00	−1.50	1.0	−3.75	ABT^o^	497	67	380	0	0.5	Sister with KCN	Eye rubber
19						L	−3.00	−2.00	1.0	−4.00	ABT	511	70	391	0	0.5		

20	2008 [7]	1	1	25	M	R	−5.75	−1.75	0.8	−6.63	I-S ~4	500	70	380	0	60		

	Total	20	13	—	*M* = 10 *F* = 3	*R* = 10 *L* = 10	—	—	—	—	—	—	—	—	—	—		
	Mean	—	—	34.8	—	—	−4.75	−2.31	0.8	−5.91	—	494	80	362	—	21.62		
	SD	—	—	±6.0	—	—	±2.78	±1.17	±0.2	±2.87	—	±20	±35	±45	—	±20.7		
	Min	—	—	22	—	—	−12.00	−4.50	0.3	−12.75	—	441	30	285	—	0.3		
	Max	—	—	42	—	—	−1.50	−0.50	1.0	−2.00	—	520	142	415	—	60.0		

^a^Best spectacle-corrected visual acuity; ^b^spherical equivalent; ^c^pachymetry; ^d^residual stromal bed; ^e^number of enhancements; ^f^keratoconus; ^g^form fruste keratoconus; ^h^skewed radial axis; ^i^inferior-superior value; ^j^central steepening; ^k^symmetric bowtie; ^l^bilateral penetrating keratoplasty; ^m^inferior steepening; ^n^left eye keratoconus status post penetrating keratoplasty; ^o^asymmetric bowtie. ^∗^Measured RSB.

**Table 4:** Risk parameters for post-PRK ectasia in all eyes (eyes from the study cohort and eyes from the literature search).

	Eye number	Age (years)	SEQ^a^ (D)	Topographic findings	Measured pachy^b^ (*μ*m)	Ablation depth (*μ*m)	Calculated RSB^c^ (*μ*m)	Retreat^d^	ERSS^e^ criteria^15^ score							Percent tissue altered (PTA) (%)
									Age	RSB	Pachy	SEQ	Topo	Total	Risk	
Literature search	1	41	−5.75	KCN^f^ or FFKCN^g^	—	—	—	1	0	—	—	0	4	—	—	—
	2		−6.75	KCN or FFKCN	—	—	—	1	0	—	—	0	4	—	—	—
	3	34	−12.75	Normal	—	144	—	1	0	—	—	3	0	—	—	—
	4	39	−5.25	KCN	—	—	—	—	0	—	—	0	4	—	—	—
	5	22	−2.00	D sign	495	32	413	—	2	0	2	0	3	7	High	16.6
	6		−2.00	SRAX^h^ −60°, I-S^i^ −0.9 (Susp.FFKCN)	495	30	415	—	2	0	2	0	3	7	High	16.2
	7	37	−2.75	I-S ~3	472	53	369	—	0	0	3	0	4	7	High	21.8
	8		−5.50	Cent. steep.^j^	441	100	291	—	0	1	4	0	4	9	High	34.0
	9	40	−6.63	SBT^k^	509	98	365^∗^	—	0	0	2	0	0	2	Low	29.1
	10		−7.25	SBT	508	106	356^∗^	—	0	0	2	0	0	2	Low	30.7
	11	42	−9.88	SBT	477	142	285	—	0	1	3	1	0	5	High	40.3
	12		−9.25	SBT	481	133	298	—	0	1	2	1	0	4	High	38.0
	13	38	−8.50	Inf. steep.^l^	520	—	—	—	0	—	0	1	3	—	—	—
	14		−8.00	Inf. steep.	510	—	—	—	0	—	2	0	3	—	—	—
	15	31	−5.38	SBT	487	—	—	—	0	—	2	0	0	—	—	—
	16	31	−4.00	Inf. steep., SRAX	492	91	351	—	0	0	2	0	3	5	High	28.7
	17	38	−2.13	Inf. steep.	509	50	409	—	0	0	2	0	3	5	High	19.6
	18	35	−3.75	ABT^m^	497	67	380	—	0	0	2	0	1	3	Moderate	23.5
	19		−4.00	ABT	511	70	391	—	0	0	0	0	1	1	Low	23.5
	20	25	−6.63	I-S ~4	500	70	380	—	2	0	2	0	4	8	High	24.0

Study cohort	1	21	−2.00	ABT, SRAX	441	44	347	—	3	0	4	0	3	10	High	21.3
	2	25	−3.88	ABT	481	71	360	—	2	0	2	0	1	5	High	25.2
	3	33	−2.75	I-S ~2	496	45	401	—	0	0	2	0	3	5	High	19.2
	4	18	−3.00	I-S ~2	535	52	433	—	3	0	0	0	3	6	High	19.1
	5		−3.63	I-S ~2	538	63	425	—	3	0	0	0	3	6	High	21.0
	6	21	−0.75	ABT	500	23	427	—	3	0	2	0	1	6	High	14.6
	7		−1.25	ABT	504	29	425	—	3	0	2	0	1	6	High	15.7
	8	39	−3.38	SRAX	552	74	428	—	0	0	0	0	3	3	Moderate	22.8
	9	24	−3.50	I-S ~1.3, SRAX	514	67	397	—	2	0	0	0	3	5	High	22.5

Total	Mean	31.7	−1.64		499	65	383		0.9	0.1	1.8	0.3	2.3	5.5		23.2
	SD	±7.6	±1.25		±26	±32	±45		±1.2	±0.4	±1.2	±0.6	±1.5	±2.3		±7.0
	Min	18	−12.00		441	23	285		0	0	0	0	0	1		14.6
	Max	40	−0.75		552	142	433		3	1	4	3	4	10		40.3

^a^Spherical equivalent; ^b^pachymetry; ^c^residual stromal bed; ^d^number of enhancements; ^e^ectasia risk score system; ^f^keratoconus; ^g^forme fruste keratoconus; ^h^skewed radial axis; ^i^inferior-superior value; ^j^central steepening; ^k^symmetric bowtie; ^l^inferior steepening; ^m^asymmetric bowtie. ^∗^Measured RSB.

**Table 5:** The distribution of eyes according to ectasia risk score system (ERSS) scores for each risk parameter.

Parameter		Points				
		4	3	2	1	0
Topography	(*n* = 29)	20.7%	41.4%		17.2%	20.7%
RSB^a^	(*n* = 22)^∗^	0%	0%	0%	13.6%	86.4%
Age	(*n* = 29)		17.2%	17.2%	0	65.6%
Pachymetry	(*n* = 25)^∗^	8.0%	8.0%	60.0%		24.0%
SEQ^b^	(*n* = 29)	0%	3.5%	0%	10.3%	86.2%

^a^Residual stromal bed; ^b^manifest refraction spherical equivalent; ^∗^included in this analysis were eyes that had available data for the specific parameter tested.
